# Knowledge is not enough to solve the problems – The role of diagnostic knowledge in clinical reasoning activities

**DOI:** 10.1186/s12909-016-0821-z

**Published:** 2016-11-24

**Authors:** Jan Kiesewetter, Rene Ebersbach, Nike Tsalas, Matthias Holzer, Ralf Schmidmaier, Martin R. Fischer

**Affiliations:** 1 Institut für Didaktik und Ausbildungsforschung in der Medizin am Klinikum der Universität München, Ludwig-Maximilians-Universität, Munich, Germany; 2 Lehrstuhl für Entwicklungspsychologie, Ludwig-Maximilians-Universität, Munich, Germany; 3 Medizinische Klinik und Poliklinik IV, Klinikum der Universität München, Ludwig-Maximilians-University, Munich, Germany

**Keywords:** Medical problem-solving, Metacognition, Knowledge categories, Clinical reasoning, Diagnostic reasoning

## Abstract

**Background:**

Clinical reasoning is a key competence in medicine. There is a lack of knowledge, how non-experts like medical students solve clinical problems. It is known that they have difficulties applying conceptual knowledge to clinical cases, that they lack metacognitive awareness and that higher level cognitive actions correlate with diagnostic accuracy. However, the role of conceptual, strategic, conditional, and metacognitive knowledge for clinical reasoning is unknown.

**Methods:**

Medical students (*n* = 21) were exposed to three different clinical cases and instructed to use the think-aloud method. The recorded sessions were transcribed and coded with regards to the four different categories of diagnostic knowledge (see above). The transcripts were coded using the frequencies and time-coding of the categories of knowledge. The relationship between the coded data and accuracy of diagnosis was investigated with inferential statistical methods.

**Results:**

The use of metacognitive knowledge is correlated with application of conceptual, but not with conditional and strategic knowledge. Furthermore, conceptual and strategic knowledge application is associated with longer time on task. However, in contrast to cognitive action levels the use of different categories of diagnostic knowledge was not associated with better diagnostic accuracy.

**Conclusions:**

The longer case work and the more intense application of conceptual knowledge in individuals with high metacognitive activity may hint towards reduced premature closure as one of the major cognitive causes of errors in medicine. Additionally, for correct case solution the cognitive actions seem to be more important than the diagnostic knowledge categories.

## Background

Clinical experts need general and specific problem solving strategies in order to make adequate treatment decisions for their patients. Clinical problem solving (or clinical reasoning) as a skill involves different categories of knowledge as well as several cognitive abilities and is key for becoming a clinical expert [1]. Problem-solving occurs in well-known phases, described in models like the hypothetical-deductive model and pattern recognition, a process that requires the use of knowledge [2–4]. In university, the focus lies on teaching medical knowledge, in order to give the student a foundation for further clinical problem-solving when dealing with real patients [5]. According to recent studies [6] diagnostic knowledge can be categorised into three categories: Conceptual knowledge (“what information”), strategic knowledge (“how information”) and conditional knowledge (“why information”) [5]. Table 1 shows an overview of the definitions. These categories have been investigated in several studies regarding clinical reasoning of medical students and medical doctors [6–8].

**Table 1:** Diagnostic knowledge dimensions according to Schmidmaier [7], van Gog [9], Krathwohl [10]

Knowledge dimension	Definition	Examples of knowledge
Conceptual Knowledge - what-	The basic elements one must know to be acquainted with a discipline or solve problems in it.	Knowledge of terminology, specific details, and elements.
Strategic Knowledge - how -	How to execute something; methods of inquiry, and criteria for using skills, algorithms, techniques and methods.	Knowledge of subject-specific skills and algorithms, subject-specific techniques and methods, and criteria for determining when to use appropriate procedures. Knowledge about problem solving
Conditional Knowledge - why -	The interrelationships among the basic elements within a larger structure that enable them to function together.	Knowledge of classifications and categories, principles and generalizations, theories, models, and structures. Knowledge about the rationale behind.
Metacognitive Knowledge - selfcognition-	How to think about thinking; knowledge about cognitive tasks, and self-knowledge	Knowledge about cognition in general as well as awareness of one’s own knowledge. Knowledge about one’s own cognition

The Revision of Bloom’s Taxonomy added a fourth category: Metacognitive knowledge, which “involves knowledge about cognition in general as well as awareness of one’s own knowledge about one’s own cognition” [9, 10]. While handling a case, medical students or doctors are able to externalize their thoughts about the strategies of problem-solving or their application of knowledge [11]. Metacognition in this sense includes the judgements of how easily one believes one learns and whether one has the feeling of knowing something.

Surprisingly, little is known about the assessment and applicability of metacognition within the medical context and its relation to the knowledge categories in the situated learning contexts of medical students.

Whereas several methods are used to assess “classic” knowledge categories (e.g. multiple choice tests, key feature problems, interviews, questions, stimulated recall) it has proven difficult to measure and observe metacognition in a realistic setting [7]. Since metacognition cannot be observed directly in students [12], self-report methods like questionnaires, rating scales and stimulated recall are used. However, these self-reporting measures already reflect that, to be able to talk what one thinks, the student’s metacognitive activities *and* one’s verbal capacity are of importance [13]. When students are thinking aloud, registering the metacognitive activities without the student’s awareness is possible and the otherwise implicit cognitive processes can be observed [14].

In clinical problem solving research, traditionally only little parts of knowledge are investigated in relation to the correct diagnosis. Thus far, there is no model of clinical reasoning that, if applied, can explain how and why successful students come to the correct diagnosis, while unsuccessful students do not. However, it seems worthwhile to create evidence for such a holistic model of clinical problem solving of medical students that should include all knowledge categories. We therefore set out to observe all aforementioned knowledge categories simultaneously in order to identify the relationship between them. More specifically we wanted to answer the following research questions:

1. How are diagnostic knowledge categories interrelated?
  The interplay of knowledge categories gives insight how students store clinical knowledge and whether some categories seem more important to them than others. Further, it has not been investigated how knowledge categories relate to previous knowledge.
2. How is the use of the diagnostic knowledge categories related to time on task?
  It is important to understand how much time the application of the different knowledge categories takes.
3. How is the use of diagnostic knowledge categories related to diagnostic accuracy?
  Especially, it seems interesting to identify the role each plays to solve a clinical case.
4. How are the knowledge categories divided over the course of a case solution?
  It is interesting to see if some of the knowledge categories are used more frequently in the beginning and others are used more towards the end of the case solutions.

To answer this research questions we conducted a study where medical students first received a short knowledge training for clinical nephrology and a subsequent knowledge test to standardize previous knowledge. After that the students worked on paper-based, clinical case scenarios while thinking a-loud. The think-a-loud protocols were transcribed and coded according to the aforementioned knowledge categories. In the following paragraphs each step of the methodology is explained in detail.

## Methods

### Participants

Twenty-one medical students (female = 11) of two German medical faculties in their third, fourth and fifth year (M = 23.9 years; range 20–34) volunteered to take part in the study. These curricular years were chosen because the participants would have finished their internal medicine curriculum and should have enough prior knowledge to solve clinical problems but would not have experienced the final sixth clinical year of full-time electives that usually elevates students’ problem-solving substantially. This study was approved by the Ethical Committee of the Medical Faculty of LMU Munich. Written, informed consent was obtained from all participants and all participants received a small monetary compensation for participation.

### Coding scheme

A coding scheme was established on the foundation of the knowledge type definitions [7, 10, 15]. The definition used in the coding scheme is illustrated in Table 2. The coding scheme had an overall interrater reliability of *k* = .79; SD = .9 for the categories. One investigator (R.E.) coded all transcripts; a random 10% sample of the text was double coded.

**Table 2:** Operationalized definition of the diagnostic knowledge dimensions

Dimension	Operationalized definition and examples
Conceptual knowledge, “what”- information	Statements of facts, repeated information. Causal knowledge or deductive reasoning without explanations.
	*Examples: “Leucocytes of 2000? The reference value was approximately 10,000?”; “Antibiotics can cause red urine as well”; “Nephrotic syndrome consists of proteinuria, hypalbumin- and dislipidaemia and edema”.*
Strategic knowledge, “how”-information	Knowledge about actions. Explanations, why one prefers this action. Strategic use of concepts.
	*Examples: “May I have an ECG?”; “First, I would like to know how many cigarettes he consumes”; “Do we have a urine sample? Since it is a cheap and quick investigation, we should do that”; “One could make an ultrasound scan in order to identify free fluid”.*
Conditional knowledge, “why”-information	Relationships between facts. Inductive reasoning, several facts are taken together in order to derive a judgement. Explanations of concepts without strategic use of those concepts.
	*Examples: “He has a cirrhosis of the kidney, and he already has anaemia and diabetes. Taken together, he has chronic kidney failure”; “Chronic kidney failure – due to this, the RAAS is activated causing the hypertension. This is the reason why medication doesn't help”.*
Metacognitive knowledge “selfcognition” -information	The meta-level (metacognition) receives information from object-level (case-work). Consciousness about information and state of cognitions. Summaries and assessment of information, self-assessment, comparison of new information with the mental representation of the case. The meta-level (metacognition) modifies the object-level (case-work). Intervention into the process of working on the case. Something changes, or not, but with intention.
	*Examples: “What have we got so far?”, “I think this is correct. I'm not completely sure, but I think it is okay”; “Oh, there really is blood in the urine, as I had assumed before”*

### Course of study

Students arrived and first filled out a pre-study questionnaire (see below), then students received a three hours of practicing a standardized learning unit in the field of clinical nephrology and upon completion, the students’ retention of content specific medical knowledge was tested using a multiple choice test. Then participants were instructed on the think-aloud method in a short practice exercise. Finally, students then solved three cases in clinical nephrology with the think-aloud method (see below).

**Fig. 1:**
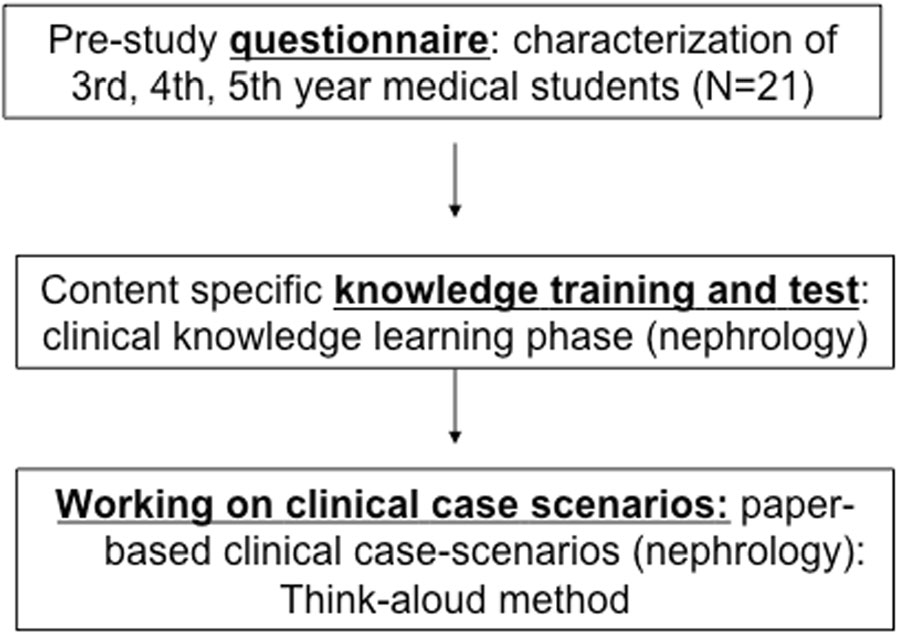
Overview of the study

Figure 1 shows the course of the study with knowledge training, a subsequent knowledge test, and work on the paper-based, clinical case scenarios.

All students were recorded and recordings were transcribed and coded according to the defined knowledge categories. Codings were analysed for accuracy of the diagnosis.

### Pre-study questionnaire

All participants completed a questionnaire containing items about their socio-demographic data, gender and age to control possible confounders. Further the participants were asked their overall grade of the preliminary medical examination. The reliability of this national multiple-choice exam is very high (Cronbachs α = .957) [16]. The performance of participants in this exam was used as an indicator for general prior knowledge in medicine. The results of the questionnaire and all other obtained data were pseudonymized.

### Knowledge training and test

Although all participants had successfully passed their internal medicine curriculum a standardized learning tool was provided to refresh the textbook knowledge. Thirty flashcards were used containing 98 items with factual information on clinical nephrology and more precisely to acute renal failure and chronic renal insufficiency. This content matches with the pathomechanisms of the used cases. The content of the flashcards was previously published in another study (appendix S1 (online) of Schmidmaier et al. [17]). Within a 3 h electronic learning module it was ensured by testing that all participants could retrieve the contents of each flash card at least once. This was to help ensure that all students were able to show their problem-solving strategy and ability because they had the knowledge needed for application of strategies.

### Clinical case scenarios

The three, paper-based case scenarios within the field of clinical nephrology were real cases from the department of internal medicine adapted by experts with anonymized, real supplemental material (i.e. lab values). After the transformation into paper-based scenarios, the cases were additionally reviewed by two content experts and one expert of medical education to ensure best possible authenticity of a paper-based case. All cases were structured the same way, containing two or three pages describing the patient’s symptoms and medical history. The results of the physical examination, blood tests, urine sample, ECG, and ultrasound scan were each described on separate pages.

The students’ task was to work on each case to show their problem-solving abilities with no instructions being given other than “Please work on this case”. They were not explicitly asked to state a diagnosis. Only one student and the test instructor were present in the room during the case elaboration. The test instructor sat behind the participant to avoid any diversion of thought [18]. The only interaction between the participant and instructor was when the instructor provided the next page of a case upon the participant’s request. Every case was interrupted after 10 min, independent of whether the case was solved or not. While participants were working on the cases using the think-aloud method, they were audio-recorded. All students did voluntarily state a diagnosis at the end of each case.

### Data analysis

All audio recordings (total time of over 12 h) were transcribed and coded using the operationalized definitions of knowledge categories and metacognition described above. Data of three case sessions of 21 participants were evaluated and 63 sessions were analysed.

The standard qualitative content analysis [19] was used to assess, code, and analyse the process of thought, as it also yields very detailed quantitative data in consecutive analysis. It uses models with several categories for the coding of a text. In this study, the knowledge categories were used. The shortest section of text matching a particular knowledge category was determined as an episode. When different knowledge categories took place at the same time, one text section could be coded as more than one category. For examples see Table 2.

Subsequently, the codings were marked as sections in the transcription software “f4” (f4 2011, Dr. T. Dresing, http://www.audiotranskription.de) and exported to Microsoft Excel 2010 (Microsoft, 2010). For further analysis, the statistical environment “R” was used (http://www.r-project.org/).

A predefined alpha level set at *p* < .05 was used for all tests of significance. If the data was used multiple times for comparisons we Bonferoni corrected for alpha error accumulation and report results as significant accordingly. Graphical illustrations were processed as the percentage of time spent on one knowledge category relative to the overall time. Although the categories of the model were described qualitatively, this was the basis for a quantitative analysis and graphical illustration of the results.

The frequencies of the categories and length of the episodes were analysed as quantitative dependent variables. The accuracy of diagnosis was established in a binary form (*correct* or *not correct*) as a dependent variable. Chi-squared tests were used to verify the relationship of dependent variables to all dichotomous socio-demographic participant variables (like gender), while Pearson correlation was used for all continuous dependent variables to correlate them to previously obtained participant data. Correlations between two dichotomous variables were calculated using crosstabs correlation coefficient ϕ. To gain insight how knowledge categories are divided over the course of time the cases were divided in 6 timewise equal parts. Frequencies of knowledge categories per sixth of the case were analysed as frequencies. As there a so many possible comparisons between the categories and sixth, we chose not to apply nonparametric statistical tests because of a to high alpha error accumulation.

## Results

### Descriptive data

**Fig. 2:**
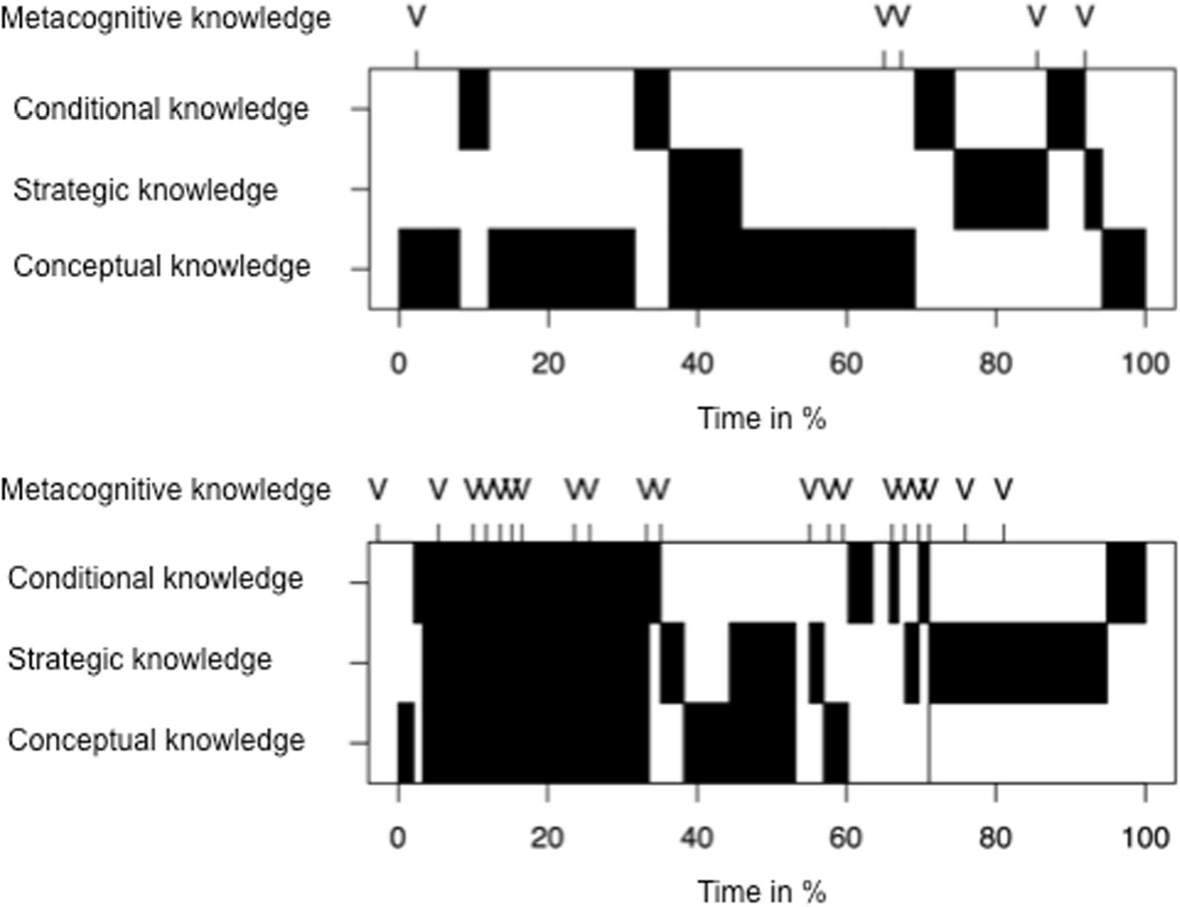
Time-line graph (Gantt-charts) of two participants of a session with a clinical case. The Gantt-chart shows the distribution of the use of different diagnostic knowledge categories over time. As metacognitive knowledge was only in use in combination with other knowledge categories its use is presented additively on top. The upper part of the figure shows a participant with only little use of the knowledge categories and the lower part of the figure a participant with much use of the knowledge categories

Overall 983 distinct episodes of knowledge categories were be coded. Table 3 shows that the students’ reasoning consists mainly of conceptual and strategic knowledge. All cases contain these categories. Most often conceptual knowledge was used (CcK) with a 44% frequency, conditional knowledge (CdK) was used with a 36% frequency, strategic knowledge (SK) with a 21% frequency. Metacognition was identified most frequently (58%) but always in combination with other knowledge categories. Metacognition was used in every case with a mean of M = 9.02 per case (SD = 6.21). Figure 2 shows the time-line graphs of two participants, exemplifying little and extensive use of metacognition.

**Table 3:** Descriptive data of diagnostic knowledge dimensions used by medical students during the cases

Knowledge dimension	Frequency	Percent
Conceptual knowledge (CcK)	432	44%
CcK only	325	33%
With other knowledge dimensions	107	11%
Strategic knowledge (SK)	349	36%
SK only	279	28%
With other knowledge dimensions	70	8%
Conditional knowledge (CdK)	202	21%
CdK only	121	12%
With other knowledge dimensions	81	9%
Metacognitive knowledge (MK)	568	58%
MK only	0	0%
With other knowledge dimensions	568	58%

Percent refer to the overall use of knowledge dimensions (CcK, SK, and CdK equal 100%)

1. How are the diagnostic knowledge categories interrelated?
  To answer this research question the frequency per case of the use of knowledge categories was correlated. Results show that conceptual and strategic knowledge are not significantly related (r_CcK;SK_ = .23;n.s.; r_CcK;CdK_ = .00;n.s.). Conceptual knowledge and metacognitive knowledge (r_CcK;MK_ = .35) are significantly related, as are conditional and strategic knowledge (r_CdK;SK_ = .27). The results are presented in Table 4.

**Table 4:** Pearson’s Correlations of the use of diagnostic knowledge dimensions

Knowledge dimension	Strategic knowledge	Conditional knowledge	Metacognitive knowledge
Conceptual knowledge	.23	.00	.35*
Strategic knowledge		.27*	.09
Conditional knowledge			.15

Significant results (*p* < .05) are marked with an asterisk
  Interestingly prior knowledge (grades of PME and assessment of the learning phase in the field of clinical nephrology) was significantly correlated to metacognitive knowledge (r_MK;PME_ = .41, r_MK; LEARNING PHASE_ = .28).
2. How is the use of diagnostic knowledge categories related to time on task?
  To answer this research question the time-on-task (TT) was correlated with the use of knowledge categories. In three cases the students had to be interrupted after 10 min. These students were included in the analysis with the maximum time. The overall time-on-task was not correlated with diagnostic accuracy (r_TT; DIAGNOSTIC ACCURACY_ = -.13;n.s.). However, conceptual and strategic knowledge is significantly correlated to TT (see Table 5).

**Table 5:** Pearson’s correlations knowledge dimensions and time-on-task

Knowledge dimension	Correlation with time-on-task
Conceptual knowledge	.27*
Strategic knowledge	.35*
Conditional knowledge	.16
Metacognitive knowledge	.24

**Fig. 3:**
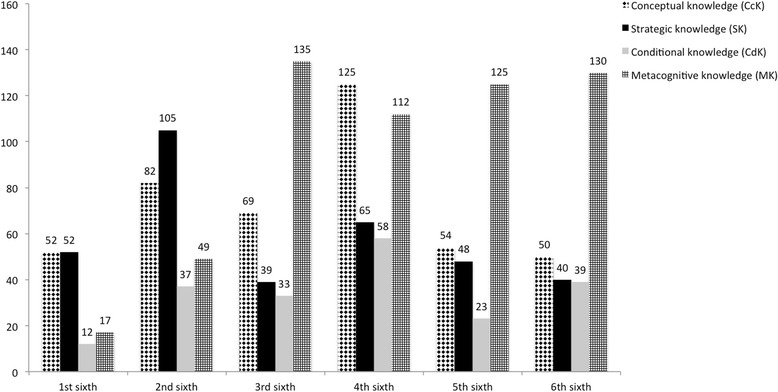
Diagnostic knowledge dimensions used by medical students over the course of the cases
  We found the occurrence of a pattern of conditional and strategic knowledge right before the closure of cases, named sequence-at-closure (s@c). This sequence-at-closure appeared in 24 of the 63 case solutions (=38%) and is significantly correlated with the correct solution of the case (r_ϕ_. _S@C; CORRECT SOLUTION_ = .37).

Significant results (*p* < .05) are marked with an asterisk
3. How is the use of diagnostic knowledge categories related to diagnostic accuracy?
  When correlating the use of the four knowledge categories to the correct solution none of them showed a significant result. As well, Chi squared tests of socio-demographic data of the participants (age, year of studies) and correct versus incorrect diagnosis yielded no significant result.
4. How are the knowledge categories divided over the course of a case solution?
  We found that frequencies of the used categories are not equally distributed over the case. Interestingly, in the first two sixth of the case the students used more conceptual and strategic knowledge. From the third sixth the students used more metacognition than any other category. Of course, metacognition could only be coded together with other categories, so there is a dependency of this category. However, the frequencies of conceptual and strategic knowledge decline in the fifth and sixth sixths. All frequencies over the course of the cases are depicted in Fig. 3.

## Discussion

In this study the different knowledge categories including metacognition in case work of medical students were empirically coded and described. The diagnostic knowledge categories were applied for the first time to medical students problem-solving in a realistic environment. The result was application of conceptual, strategic and conditional knowledge throughout the cases. None of the knowledge categories on its own has a crucial role for good performance. Further, prior knowledge was not directly related to the correct diagnosis. These results supports the claim that it is not simply knowledge which solves clinical cases and more in this sense does not directly mean better. Instead, it is the goal-directed application of knowledge in a certain order that helps to solve cases. Over the course of the cases it seems that the application of conceptual and strategical knowledge declines, while the importance of metacognitive knowledge increases. We found that oftentimes the last two categories before a diagnostic decision was made by the participants consisted of a pattern of conditional and strategic knowledge at the closure of cases, named sequence-at-closure, which correlated with the correct solution of the case. This result relates to our previous findings regarding the so called higher loop of cognitive actions, which was associated with better diagnostic performance [20]. The higher loop consisted of the cognitive actions Evaluation, Representation and Integration. It seems that students who are ready to state a correct diagnosis evaluate and summarize their represented knowledge about the case with this final pattern of conditional and strategic knowledge before integrating into the correct solution. If students have a clear representation of the case in relation to their predefined clinical knowledge they know, why the patient’s symptoms and clinical findings occur and how to deal with them, then they have a very good chance to correctly diagnose the patient. This finding has direct implications for instructional medical education research, which we will discuss further below.

Metacognition could be coded in all participants. However, it always appeared in conjunction with other knowledge categories. This result seems plausible as the application of metacognition cannot be separated from the content of a case. The coding of metacognition was worthwhile; it significantly correlated with conceptual knowledge and with two distinct measures of prior knowledge. People who know more and scored better in their previous studies seem to have additional capacity to control and monitor their solution in a better way. Knowledge regularly is measured in assessment and learning research [6]. Thus far only a few studies take metacognitive knowledge into account. The few available studies take into regard interventional aspects, namely reflective practice [21–23]. There are many ways to assess metacognition. With our method, we tried to go one step beyond the current approaches to understand what is happening in the mind of medical students. It shows that high and low performers are not distinguished simply by their use of knowledge categories.

### Limitations of the study

Our study has several limitations. First of all, aside from the correct solution of the case we did not code the performance within the knowledge categories. The knowledge could contain incorrect explanations and procedures. However, thus far there is no study that shows that the student who arrives at a correct diagnosis can necessarily only deduct it from correct knowledge.

The study included 21 participants and three cases per participant. We are aware that this sample is limited; this was necessary due to the elaborate data preparation process. On the other hand, qualitative research chooses to focus on the phenomenon of interest to unfold naturally, rather than a controlled influence of the interplay of variables [24]. The sample is relatively large considering it is a qualitative approach. The paper-based cases, while constructed with the most care and best possible authenticity, are still cases and not real patients with a real patient encounter with gestures and appearance and the possibility to ask the patient additional information. Thus the transferability to an authentic clinical environment might be limited. The think-aloud method limits our findings in a way that only verbal expressions can be analysed further coded and thus interpreted. Talking during the thought process requires metacognitive ability and this does confound with our dependent variable. Therefor, if some participants were more talkative than others they could possibly provide more information in all categories. However, we did not find any significant correlation between number of words expressed and number of categories coded.

The students who took part in our study volunteered and thus we cannot exclude a selection bias. The PME scores that we obtained, however are spread equally over the passing grades from 65 to 87% (M = 77.1%; SD = 6.6) and do not differ from the rest of the cohort of students.

## Conclusions

The findings presented here show that the use of knowledge is not enough to distinguish between high and low performers. Further, it shows that the time students spent on the task is neither a positive nor a negative predictor for diagnostic accuracy. When medical educators design interventions to foster clinical reasoning it is important not to focus too much on the use of specific knowledge categories, but teach the use of the right sequences of knowledge at the right time, including the application of metacognition. This goes in line with a renewed conceptualization of the term “script” where students are supposed to go beyond the illness of the patient, but see diagnostic actions as a stereotypic process in which they learn the content of illnesses [25]. Studies investigating the interplay of cognitive actions and knowledge categories with instructional methods such as self-explanation prompts [8, 21, 26, 27] are a promising next step in the endeavour to understand and foster clinical reasoning in medical students.
